# Effects of Potato Protein Isolated Using Ethanol on the Gelation and Anti-Proteolytic Properties in Pacific Whiting Surimi

**DOI:** 10.3390/foods11193114

**Published:** 2022-10-06

**Authors:** Won Byong Yoon, Jae Won Park, Hwabin Jung

**Affiliations:** 1 Department of Food Science and Biotechnology, College of Agriculture and Life Sciences, Kangwon National University, Chuncheon 24341, Korea; 2 Elderly-Friendly Food Research Center, Agriculture and Life Science Research Institute, Kangwon National University, Chuncheon 24341, Korea; 3 OSU Seafood Research and Education Center, Oregon State University, Astoria, OR 97103, USA

**Keywords:** potato protein isolate, Pacific whiting surimi, protease inhibition, autolysis, gel texture

## Abstract

Pacific whiting is a primary species utilized for surimi processing in the Pacific Northwest of the US. However, endogenous protease in Pacific whiting surimi deteriorates the quality during slow cooking. The demand for clean-labeled and economically competitive protease inhibitors has been increasing. In the present study, the anti-proteolytic effect of potato protein isolate (PPI), a by-product from the potato starch industry, prepared using 20% ethanol on the endogenous protease activity of Pacific whiting (PW) surimi was investigated. The ohmic heating method was carried out for a better assessment of the anti-proteolytic activity of inhibitors. A factorial design was carried out in which the independent variables were the four types of inhibitors and their concentration (0, 0.5, 1, 2, and 3% *w*/*w*) at two heating conditions. The heating condition was used as a blocking factor. All experiments were randomized within each block. The addition of 2% PPI which demonstrated the highest anti-proteolytic activity among five different concentrations significantly increased the breaking force, penetration distance, and water retention ability of PW surimi gel as the endogenous proteases were effectively inhibited when heated ohmically at 60 °C for 30 min prior to heating up to 90 °C. In addition, SDS-PAGE disclosed that PPI successfully retained the intensity of myofibrillar heavy chain (MHC) protein of PW surimi gels even under the condition at which proteases could be activated at 60 °C. The whiteness of gels was not negatively affected by the addition of PPI. Comparing all samples, a denser and more ordered microstructure was obtained when PPI was added. A similar trend was found from the fractal dimension (*Df*) of the PPI-added gel’s microstructure. Therefore, PPI could be an effective and non-allergenic protease inhibitor in PW surimi leading to retaining the integrity of high gel quality.

## 1. Introduction

Pacific whiting is one of the major resources in the Pacific Northwest of the United States which is primarily utilized for surimi processing. However, Pacific whiting surimi undergoes textural softening at a temperatures around 50–60 °C, due to its endogenous protease activity when cooked slowly [1,2]. The major protease in Pacific whiting is cathepsin. Cathepsin B and H were found in Pacific whiting fillet, but approximately half of cathepsin B and entire cathepsin H are removed during the washing steps of surimi processing. However, entire cathepsin L is not removed during washing, causing degradation of myofibrillar proteins and inhibiting optimal gel formation upon slow heating such as in water bath cooking [3,4].

Protein additives have been used in surimi to reduce the degradation of myofibrillar proteins by endogenous proteases. Potato extract, whey protein, legume proteins, various plasma proteins (chicken, beef, pork, or salmon), and egg white have been used as food-grade protease inhibitors in surimi [5,6,7,8,9,10]. However, these inhibitors have drawbacks due to various reasons (bovine spongiform encephalopathy, allergy, high price, odor, off-color, or off-flavor) [11]. Therefore, plant proteins are likely to be suitable for use in the surimi industry based on their safety and competitive price.

The potato starch industry discharges wastewater, which is a by-product called potato fruit juice (PFJ). PFJ contains 2–5% solids, of which crude protein represents about 35%. The PFJ crude protein comprises proteins, peptides, amino acids, and amides. The major soluble potato proteins are found to be the protease inhibitors (4–25 kDa) and patatin (39–45 kDa), which comprise 38% and 50% of the crude proteins, respectively [12,13]. Potato protein recovered from PFJ has high nutritive values and functional abilities such as emulsifying and anti-protease activity. The utilization of potato protein as a food ingredient may be challenging because isolating proteins from PFJ can affect their functional properties negatively [14]. However, since potato protein is much less common in allergies, it can be a successful replacement for egg white, gluten, soy proteins, fish proteins, and nut proteins in food formulations [15].

To better understand the effect of protease inhibition on the gelation properties of surimi, ohmic heating has been used as a uniform and linear heating method [16,17]. In addition, the microstructure of protein gels has been expressed and quantified with fractal characteristics [18,19]. Therefore, our objectives were 1) to apply 0–3% isolated potato proteins to Pacific whiting surimi and to evaluate their effect on gelation under different ohmic heating conditions, one method that directly heats the gel to 90 °C and another method that activates protease at 60 °C prior to heating to 90 °C, and 2) to compare the protease inhibitory effect among the isolated potato proteins and the same amount of commercial protease inhibitors on physical, chemical, and microstructure properties in Pacific whiting surimi.

## 2. Materials and Methods

### 2.1. Materials

Pacific whiting surimi containing cryoprotectants (4% sugar, 5% sorbitol, and 0.3% sodium polyphosphate) with 74.3% (w.b.) moisture content was obtained from a local surimi plant (Pacific Surimi, Newport, OR, USA) and kept at −30 °C. Fresh potatoes of the Russet Burbank variety were purchased from a local supermarket and kept at 4 °C until used. The commercial protease inhibitors, egg white and dry potato extract X-TEND™, were provided by Michael Foods (Minnetonka, MN, USA) and Basic American Foods (Moses Lake, WA, USA), respectively. All chemicals used in this study were of analytical grade.

### 2.2. Preparation of Potato Fruit Juice (PFJ)

PFJ was prepared according to the modified method of Vikelouda and Kiosseoglou [20] and Waglay et al. [14]. Fresh potatoes were washed, peeled, and chopped into small pieces (1 cm cubes). The potato pieces were blended with an equal amount of distilled water containing 0.5% ascorbic acid, and then the slurry was filtered through a double-layered cheesecloth before centrifuging at 8000× *g* at 4 °C for 30 min using a Beckman J6-MI centrifuge (Beckman Coulter, Fullerton, CA, USA). The supernatant filtered through a filter paper (No. 44, Whatman, Maidstone, Kent, UK) was collected. The clear yellowish liquid was treated as a simulated PFJ. The prepared PFJ was freeze-dried (FreeZone 12L, Labconco Corp., Kansas City, MO, USA) and used for further analysis to compare its protease inhibitory effect with the other inhibitors used in surimi gels.

### 2.3. Potato Protein Isolation

The protein isolation of potatoes using ethanol was performed according to the method of Waglay et al. [14]. Firstly, ethanol (95%) was added to the PFJ at a ratio of 20% (*v*/*v*). Then the mixture was stirred at 100 rpm at 4 °C for 1 h before centrifuging at 8000× *g* at 4 °C for 30 min. The ethanol-treated precipitates were freeze-dried and stored at −20 °C as PPI (potato protein isolate).

### 2.4. Effect of Inhibitors on Protease Inhibition

The protein concentration of various protease inhibitors [potato extract (PE), egg white (EW), freeze-dried PFJ (FD-PFJ), and PPI] was measured using a Bio-Rad protein assay kit (Bio-Rad Laboratories, Hercules, CA, USA) based on the Bradford method [21]. The inhibitors were also tested for endogenous protease inhibition activity in Pacific whiting surimi following the same procedures used in the study of Fowler and Park [1]. Three grams of partially thawed Pacific whiting surimi were mixed with the inhibitors in a dry powder form at various concentrations (0, 0.5, 1, 2, and 3% *w*/*w*). The samples were incubated at 55 °C in a temperature-controlled water bath for 60 min, while the blank samples were placed in ice water for 60 min. The autolytic activity was terminated by the addition of 27 mL of 5% cold trichloroacetic acid (TCA). The samples were homogenized using a tissue homogenizer (Tissue Tearor Homogenizer, Biospec Products Inc., Bartlesville, OK, USA) for 1 min, followed by incubation at 4 °C for 15 min. The homogenized samples were centrifuged at 8000× *g* for 10 min. TCA-soluble peptides in the supernatant were determined by the Lowry method [22] using L-tyrosine as a standard.

(1) $$\%\;\mathrm{inhibition}=\frac{\left(TC_{55}-TC_{0}\right)-\left(TI_{55}-TI_{0}\right)}{\left(TC_{55}-TC_{0}\right)}\times 100$$

where *TC*_55_ is protein concentration of the control sample (no inhibitor added) incubated at 55 °C, *TC*_0_ is tyrosine of the control sample placed in ice water, *TI*_55_ is the protein concentration of samples treated with the inhibitors and incubated at 55 °C, and *TI*_0_ is the protein concentration of samples with the inhibitors placed in ice water.

### 2.5. Surimi Gel Preparation

The surimi pastes were formulated as in Table 1 and the gel preparations were conducted according to the method of Fowler and Park [1]. First, partially thawed surimi (at approximately −5 °C) was placed in a vacuum silent cutter (UM 5 universal, Stephan Machinery Corp., Columbus, OH, USA). The surimi was chopped at 1800 rpm for 1 min, followed by adding 2% NaCl and chopping at 1800 rpm for 1 min. Chopping continued at 1800 rpm for another 1 min with the addition of protease inhibitors in a dry powder form at various levels (0, 0.5, 1, 2, and 3% *w*/*w*) and the addition of ice to adjust the moisture content to 78%. Final chopping was conducted at 3600 rpm under vacuum (40–60 kPa) for 3 min. The temperature of the surimi paste during chopping was maintained below 15 °C using an ethylene glycol-based chiller.

The paste was stuffed into nylon tubes (15 cm in length and 3 cm in diameter), then heated using a custom-designed ohmic heater at 250 V voltage and 10 kHz frequency under two different heating conditions to investigate the effect of protease inhibition activity of the various inhibitors on the endogenous proteases in PW surimi: (1) fast heating straight to 90 °C (in approximately 30 s) at voltage gradient 16.7 V/cm to inactivate endogenous proteases before damaging myofibrillar proteins and (2) heating to 60 °C and holding for 30 min using the same voltage gradient to activate endogenous proteases prior to heating to 90 °C. The prior heating condition is advantageous for examining the effect of inhibitors as an ingredient in the formation of the surimi gel network regardless of the anti-proteolytic activity because the protease activity can be ignored due to the very short heating time. In addition, this fast heating is analogous to commercial crabstick processing in that the temperature of the thin sheet reaches 90 °C within 1 min [23]. On the other hand, the latter heating condition allows for a better assessment of surimi proteases, which results in gel softening by activating the proteases at 60 °C similarly to the slowly heated water bath cooking method. Cooked gels were placed immediately in a plastic bag and chilled in ice water for 30 min. The gels were stored at 4 °C overnight for further analysis.

### 2.6. Water Retention Ability Measurement

Water retention ability was measured using No. 44 Whatman filter papers. Two grams of finely chopped gel samples were placed on three layers of filter paper. The sample was wrapped with the filter papers and centrifuged at 2000× *g* for 10 min. Then, the sample was removed from the filter papers and weighed. The water retention ability was calculated as follows:

(2) $$\mathrm{Water}\;\mathrm{retention}\;\mathrm{ability}\;\left(\%\right)=[\frac{W_{0}-W}{W_{0}}]\times 100$$

where *W*_0_ is the initial weight of surimi gel and *W* is its weight after centrifugation.

### 2.7. Texture Analysis

Gel texture was evaluated using a texture analyzer (TA-XT plus, Texture Technologies Corp, Hamilton, MA, USA). Prior to the fracture test, the gels stored overnight at 4 °C were placed at room temperature for 2 h and cut into a cylindrical shape with a length of 2.5 cm. Breaking force (g) for gel strength and penetration distance (mm) for gel elasticity were measured using a spherical probe with a 5 mm diameter and a crosshead speed of 1 mm/s.

### 2.8. Determination of Whiteness

The whiteness of the Pacific whiting surimi gels was evaluated using L*, a*, and b* values obtained by a colorimeter (CR-310, Minolta Camera Co. Ltd., Osaka, Japan). Whiteness was calculated using the following equation:

(3) $$\mathrm{Whiteness}=100-{\left[{\left(100-L^{*}\right)}^{2}+a^{*2}+b^{*2}\right]}^{1/2}$$

### 2.9. Sodium Dodecyl Sulfate-polyacrylamide Gel Electrophoresis (SDS-PAGE)

The effect of the inhibitors on the protein patterns of Pacific whiting surimi gels was examined according to the method of Laemmli [24]. To prepare the samples for SDS-PAGE, three grams of samples were homogenized with 27 mL of hot 5% SDS (85 °C) for 1 min. After incubating the sample in a water bath at 85 °C for 1 h, the mixture was centrifuged at 8000× *g* for 20 min. The protein concentration of the supernatants was analyzed by the method of Lowry et al. [22]. The sample aliquots (protein concentration of 2.0 mg/mL) containing 25 μg protein were used for SDS-PAGE. Molecular weights of the gel samples were determined on a 4% acrylamide stacking gel and 10% acrylamide separating gel using a Mini-Protean III unit (Bio-Rad Laboratories, Hercules, CA, USA). The proteins were stained with 0.125% Coomassie brilliant blue R-250 (Bio-Rad Laboratories, Hercules, CA, USA) and de-stained in a 50% methanol with 10% acetic acid solution. The molecular weights of the samples were determined using protein standards (Protein Plus All Blue, Bio-Rad Laboratories, Hercules, CA, USA).

### 2.10. Scanning Electron Microscopy (SEM)

SEM analysis was performed in the same procedure as the study of Moon et al. [25]. Surimi gels made with 0 and 2% protease inhibitors were cut into 2 × 2 × 1 mm size and rinsed in distilled water two times for 30 min. The gels were fixed in a 0.1 mol/L cacodylate buffer containing 2.5% glutaraldehyde and 1% paraformaldehyde at room temperature (25 °C) for 2 h. The dried samples were prepared by dehydrating through serial acetone dilutions followed by critical point drying. Then the dried samples were coated with gold and palladium (40:60). The microscopic structure of the samples was observed at a magnification of 5000×. This work was carried out at 5 kV in a field emission scanning electron microscope using a Quanta 600 FEG (FEI Inc., Hillsboro, OR, USA) at the Oregon State University Electron Microscope Facility (Corvallis, OR, USA).

### 2.11. Evaluation of Fractal Dimension (Df)

The microstructure images of gels made with 0 and 2% protease inhibitors were subjected to image analysis. The images were 450 × 400 pixels in resolution. The microstructure images of surimi gels were analyzed using the software ImageJ v1.49 (National Institutes of Health, Bethesda, MD, USA) to obtain binary images, as well as to perform fractal analysis using the Frac-Lac (2015Febb5810) tool. The original images were converted to 8-bit format and then the threshold was obtained from the range of 0 to 70 by subtracting the grey level histogram values of a binary image. This binarization step allows extraction of the patterns of structural arrangements of the surimi gels by reducing noise and emphasizing features of the microstructure images. Fractal dimension (*Df*) values of the Pacific whiting surimi gels were calculated by the box-counting method, given in the following equations [19]:

(4) D=log Nε/log ε

(5) Df=D+1

where *Nε* is the number of boxes containing parts of the images at a scale *ε*.

### 2.12. Statistical Analysis

The experiment followed a factorial design, and all experiments were completely randomized in each heating condition. The heating condition was used as a blocking factor. All the analyses were performed at least five times. The results were subjected to an analysis of variance (two-way ANOVA) with subsequent post hoc Tukey’s honestly significant difference (HSD) at the significance level of 5% using the software IBM SPSS Statistics 21 (IBM Corporation, New York, NY, USA). For the fractal dimensions, Student’s *t*-test was used to compare data between the heating conditions for significant differences.

## 3. Results and Discussion

### 3.1. Effect of PPI on Autolysis of Surimi Paste

**Figure 1:**
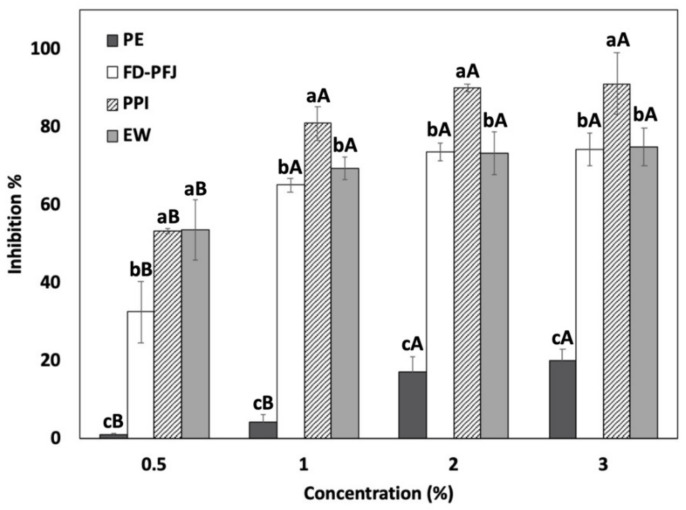
Autolysis inhibition of Pacific whiting surimi with four different inhibitors at various concentrations (PE, potato extract; FD-PFJ, freeze-dried potato fruit juice; PPI, potato protein isolate; and EW, egg white). A significant difference (*p* < 0.05) among the inhibitors with the same concentration is denoted by lowercase letters and a significant difference (*p* < 0.05) by various concentrations in each inhibitor is denoted by capital letters.

Autolysis inhibition of PPI was compared to the three other inhibitors (PE, FD-PFJ, and EW) at various concentrations in PW surimi paste (Figure 1). The soluble protein content was determined as the inhibitory proteins exist in the soluble part [14,26], and that of PE, FD-PFJ, PPI, and EW is 11.27, 29.49, 99.27, and 471.61 mg/g, respectively.

All potato-based inhibitors showed protease inhibitory activity proportional to their protein concentrations. However, the anti-proteolytic activity was not proportional to the absolute value of protein content for all inhibitors because the differences exist in the uniqueness of the functionality and fraction of soluble proteins exhibiting inhibitory activity against Pacific whiting proteases. The highest protease inhibition effect was found when PPI was added, followed by EW, FD-PFJ, and PE. The inhibition activity in the surimi paste at 2 and 3% PPI was 89.97 and 91.03%, respectively. The percentage inhibition values of the FD-PFJ and EW were less than 80%, and the inhibitory activity of PE was only 20.14% at the 3% level. This result agrees with the study of Morrissey et al. [7], that the autolysis inhibition percentage of PE in Pacific whiting surimi showed a lower value than beef plasma protein (BPP) and EW. FD-PFJ showed high autolysis inhibition percentage even with the low protein content because inhibitory proteins used as a substrate might have been less damaged due to no further processing [14]. The addition of 2% PPI showed comparable autolysis inhibition activity to salmon plasma protein (89%) and bovine blood plasma protein (90%) in PW surimi paste at 1% concentration [1,7]. In addition, the trypsin and papain inhibition activities of PPI were found higher than 90 and 95% at 2 and 3% concentration, respectively (data not shown).

### 3.2. Surimi Gel Texture and Water Retention as Affected by Various Protease Inhibitors

The texture of surimi gels with various concentrations of the inhibitors corresponded well to the result of autolytic inhibition percentage of surimi paste (Table 2 and Table 3). The addition of inhibitors in surimi gels under two different heating conditions increased the breaking force and penetration distance of PW surimi gels except for FD-PFJ. Ascorbic acid and soluble components of potato in FD-PFJ might have contributed to decreasing breaking force and penetration distance. Degradation of myosin heavy chain in PW surimi at low pH was reported in the study of Thawornchinsombut and Park [27], probably due to acid hydrolysis. For the gels ohmically heated to 90 °C, the breaking force and penetration distance of surimi gel with PPI at 1–3% levels showed the highest values among all inhibitors (Table 2). The gels containing PE showed no significant differences (*p* ≥ 0.05) at all concentrations. The texture properties of PW surimi gels with PPI and EW significantly (*p* < 0.05) increased as the concentration increased. This result indicated that the proteins of PPI and EW strengthen the heat-induced gel structures of PW surimi gel. The improvement of gel strength by PPI and EW may be due to their ability to form gel networks by heat treatment and improving interactions by acting as an enhancer in the gel [28,29,30]. The difference in enhancing the degree of breaking force and penetration distance of the PW gels with PPI and EW was likely resulted from the distinctive protein–protein interaction with the myofibrillar protein under the fast-heating condition.

For the gels held at 60 °C for 30 min prior to heating to 90 °C, the control surimi gel showed the lowest breaking force and penetration distance, as proteases were activated at 60 °C and degraded myofibrillar proteins during the holding period (Table 3). However, the addition of inhibitors significantly (*p* < 0.05) increased the breaking force and penetration distance of the gels compared to the control sample, indicating that proteins in inhibitors served well as alternative substrates to the proteases. The texture of surimi gels with 2 and 3% of PPI and EW held at 60 °C demonstrated similar values of texture properties of the control sample heated to 90 °C. However, the breaking force and penetration distance of the gels with EW at 2 and 3% showed a higher value than the gels with PPI. It appeared contradictory to autolytic enzyme inhibition measured after holding at 55 °C for 60 min (Figure 1) in which PPI showed a higher inhibition percentage than EW, due to the contribution of heat-induced gelation of EW in the surimi gels heated up to 90 °C. When surimi paste mixed with the effective inhibitors (PPI or EW) was gelled, the difference in molecular size and properties of the inhibitors might have played a certain role. The globular proteins in EW reinforce the gel network by aggregating in ovalbumin during heating, whereas small plant proteins such as legume proteins act as co-gelling agents or fillers or binders in surimi gels [31].

The water retention ability (WRA) of the surimi gels using the two different heating conditions is presented in Table 4. The water retention percentage of the control sample heated to 90 °C and held at 60 °C prior to heating to 90 °C was 49.7 and 26.8%, respectively. This indicated the endogenous proteases degraded myofibrillar proteins at 60 °C, resulting in poor network structure, causing exudation of free water by exposing hydrophilic groups [19]. The WRA corresponded to the breaking force and penetration distance of surimi gels except for the gels with PE. This result may be explained by the fiber or gelatinized starch in PE, which absorbs or adsorbs water molecules effectively. The addition of protease inhibitors prevented a loss of water by retaining moisture in their structure. The surimi gels with PPI and EW showed high WRA, which indicates the formation of a protein matrix that sufficiently imbibes water throughout the network. The highest WRA of surimi gel was observed at 2% PPI under both heating conditions. This suggested that the addition of 2% PPI effectively inhibited the proteases in PW surimi and enhanced gel quality. Potato proteins contain a number of protease inhibitors against serine and cysteine proteases. It is known that the protease inhibitor proteins in potatoes are more effective for serine proteases, whereas the remarkable anti-proteolytic activity was shown for the cathepsin L in PW surimi which is a cysteine protease in the present study [10]. However, the addition of 3% PPI may be beyond its threshold point, resulting in a decrease in water retention property.

### 3.3. Protein Patterns of Surimi Gels as Affected by Protease Inhibitors

**Figure 2:**
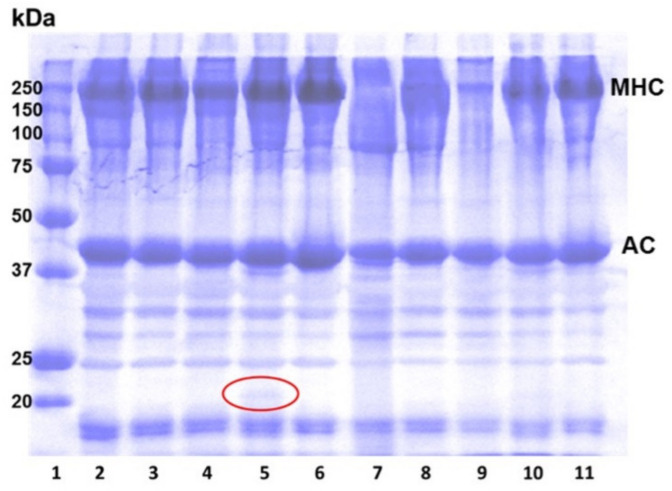
SDS-PAGE patterns of Pacific whiting surimi gels without and with 2% inhibitors. Lane 1, protein marker; Lane 2–6, the gels heated to 90 °C directly; Lane 7–11, the gels held at 60 °C prior to heating to 90 °C. Lane 2 and 7, control; Lane 3 and 8, PE; Lane 4 and 9, FD-PFJ; Lane 5 and 10, PPI; Lane 6 and 11, EW; MHC, myosin heavy chain; AC, actin.

SDS-PAGE patterns of PW surimi gels with and without 2% of inhibitors under two different heating conditions are shown in Figure 2. The high intensity of myosin heavy chain (MHC) at 205 kDa was maintained in the surimi gels heated to 90 °C (Lane 2–6). In contrast, the MHC band completely either disappeared or the band intensity was decreased for the gels prepared without any inhibitors (Lane 7) or those prepared with weaker inhibitors (PE or FD-PFJ) subjected to holding at 60 °C prior to heating to 90 °C (Lane 8 and 9). For the gels with PPI, the protein band around 20 kDa was observed (circle in Lane 5). This small protein is likely a protease inhibitor with a molecular weight ranging from 5 to 25 kDa [14]. The band around 20 kDa for the gels with PPI disappeared when the surimi gel was held at 60 °C (Lane 10), indicating PPI, as a protease inhibitor, markedly retained MHC as much as the protease inhibitors of EW including ovoinhibitor, ovastatin, cystatin, and ovomucoid (Lane 6 and 11). The small protease protein in PPI interacts with the active site of protease and forms the protease-inhibitor complex, similar to the enzyme-substrate interaction [32]. The disappearance of the protein band around 20 kDa indicates the small proteins are involved in the protease-inhibitor interaction; thus, the proteases in PW surimi gels were not active even at 60 °C. This result is concomitant with the high breaking force, penetration distance, and WRA of the gel with 2% PPI.

### 3.4. Whiteness of Surimi Gels

The whiteness of the surimi gels, affected by inhibitors and heating conditions, is shown in Table 5. When pre-treatment (60 °C) was applied to activate proteases in surimi gels before heating at 90 °C, whiteness was improved significantly regardless of inhibitors compared to the gels heated to 90 °C directly. This may be due to light scattering caused by water molecules released through the loosened gel matrix [33]. All samples demonstrated a quite good range of whiteness (approximately 76 to 82) except the gels with PE at 2 and 3% levels. However, the chroma and hue angle (Tables S1 and S2), which indicate color intensity and colorfulness, respectively, of the gels containing PE showed a minor change (Tables S1 and S2). The chroma and hue angle tended to decrease with an increase in the concentration of all inhibitors, in particular, FD-PFJ and PPI, indicating a more pale and yellowish color. Compared to EW, which is often utilized as an enzyme inhibitor in commercial surimi, PPI was very comparable in terms of whiteness, which is the critical color factor in determining the quality of surimi gel, indicating that PPI can maintain the color quality of PW surimi gels as much as EW does. The whiteness of surimi gels with certain protease inhibitors such as chicken plasma protein [8], black bean and mung bean proteins [34], yellowfin tuna roe [35], and coconut husks [36] was decreased because of the inherent color of the prepared protein powder. However, PPI gels show acceptable color quality up to 3% addition presenting similar whiteness values to the gels with EW, which is a commonly used ingredient that preserves the whiteness of surimi gels [28].

### 3.5. Microstructure and Its Patterns of Surimi Gel with Various Enzyme Inhibitors

**Figure 3:**
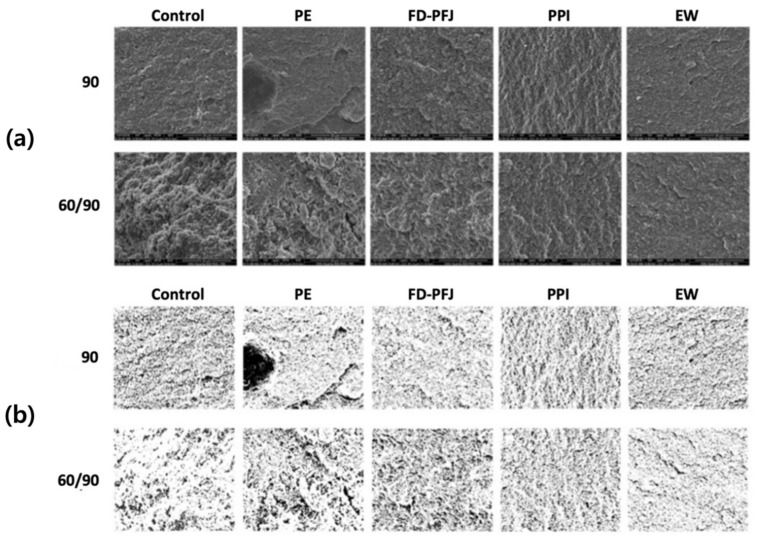
Images of the surimi gels with 2% inhibitors observed by SEM at 5000× magnification. (**a**) is microstructure of surimi gels; (**b**) is corresponding binary images of the microstructure in (**a**); 90 is the gels heated to 90 °C directly and 60/90 is the gels held at 60 °C for 30 min prior to heating to 90 °C. PE, potato extract; FD-PFJ, freeze-dried potato fruit juice; PPI, potato protein isolate; EW, egg white.

**Figure 4:**
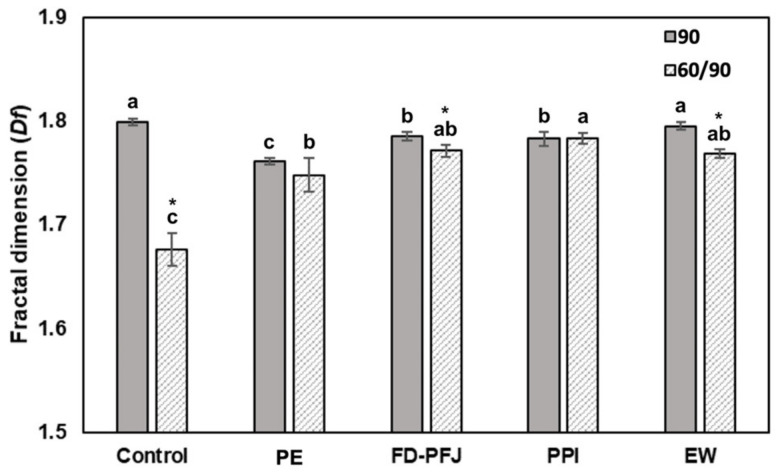
Fractal dimension (*Df*) of binary images of Pacific whiting surimi gels with different inhibitors heated to 90 °C directly (90) and held at 60 °C for 30 min prior to heating to 90 °C (60/90). PE, potato extract; FD-PFJ, freeze-dried potato fruit juice; PPI, potato protein isolate; EW, egg white. Different letters indicate a significant difference (*p* < 0.05) among the samples for each heating condition, and the symbol (*) indicates a significant difference (*p* < 0.05) between the heating conditions for each sample.

The patterns of protein aggregates in surimi gels made with 0 and 2% protease inhibitors were analyzed using microstructure obtained by SEM observation (Figure 3). The microstructure of the control, PE, and FD-PFJ gels held at 60 °C and then heated to 90 °C did not appear in the ordered structure and looked coarse. In contrast, the gel structure with PPI and EW looked dense and ordered. The microstructure of the gels can be sophisticatedly visualized; however, it is difficult to provide objective and quantitative data [37]. Therefore, the binary images of microstructure (Figure 3b) were applied to describe the irregularity of the structural arrangement of the protein with fractal dimension (*Df*) calculated by Equations (4) and (5) (Figure 4). The *Df* of the surimi gels with inhibitors was similar to the control sample heated directly to 90 °C, while the control sample held at 60 °C showed a significantly (*p* < 0.05) lower *Df* than other samples. The *Df* of PPI samples under both heating conditions was not significantly different (*p* ≥ 0.05) with the control sample directly heated to 90 °C. The addition of PPI increased the *Df* from 1.68, when pre-treatment (60 °C) was applied to activate proteases in surimi before heating at 90 °C, to 1.78. This result agrees with the study of Zhu et al. [19] that the sample pre-treated at 60 °C showed the lowest fractal dimension whereas the sample with fresh silkworm powder had the highest value. It indicated PPI inhibited proteases well in the PW surimi gels.

## 4. Conclusions

The anti-proteolytic activity of PPI was investigated and its effect on PW surimi was compared to other commercial inhibitors. PPI demonstrated a significantly high protease inhibition activity to the PW surimi protease, as shown in the autolysis inhibition measurements. The addition of 2% PPI increased breaking force, penetration distance, WRA, and retained MHC and whiteness of the PW surimi gels. When the endogenous protease of PW surimi was activated (holding at 60 °C), PPI successfully interacted with the PW protease, resulting in the disappearance of protein band around 20 kDa in SDS-PAGE which corresponds to the protease inhibitor proteins of potatoes. The PW surimi gels with PPI showed the comparable quality to the gels made with EW, which is commonly used in the industry. In addition, the dense and ordered microstructure was attained by adding PPI, showing a high *Df*. This represents that PPI was gelled within the PW surimi proteins possibly as a filler or binder. Considering EW is an allergenic ingredient, PPI has a great potential to replace it effectively in Pacific whiting surimi or other surimi in which protease activity is an issue. This non-allergenic PPI from potato by-product (PFJ) may possess a value as a competitive enzyme inhibitor and can be considered for a clean label.

## Figures and Tables

**Table 1:** Formulation of the PW surimi gels containing inhibitors.

Ingredient (g)	Inhibitor (%)				
	0	0.5	1	2	3
Pacific whiting surimi	623.1	607.5	591.9	560.7	529.6
Ice	160.9	172.5	184.1	207.3	230.4
Salt	16	16	16	16	16
Inhibitor	0	4	8	16	24
Total	800	800	800	800	800

**Table 2:** Breaking force and penetration distance of the Pacific whiting surimi gels with four different inhibitors (PE, FD-PFJ, PPI, and EW) at 0, 0.5, 1, 2, and 3% levels heated to 90 °C directly.

Concentration (%)	Breaking Force (g)				Penetration Distance (mm)			
	PE	FD-PFJ	PPI	EW	PE	FD-PFJ	PPI	EW
0	262.8 ± 9.8 ^a^	262.8 ± 9.8 ^c^	262.8 ± 9.8 ^e^	262.8 ± 9.8 ^d^	11.3 ± 0.4 ^a^	11.3 ± 0.4 ^b^	11.3 ± 0.4 ^c^	11.3 ± 0.4 ^c^
0.5	256.9 ± 11.2 ^aD^	338.0 ± 18.8 ^bA^	319.4 ± 15.2 ^dB^	279.5 ± 14.8 ^cC^	10.8 ± 0.3 ^bB^	12.0 ± 0.6 ^aA^	11.9 ± 0.5 ^bA^	10.8 ± 0.3 ^dB^
1	268.4 ± 15.7 ^aC^	379.9 ± 17.6 ^aA^	382.3 ± 18.2 ^cA^	309.2 ± 15.5 ^bB^	10.9 ± 0.4 ^bD^	12.4 ± 0.3 ^aB^	13.1 ± 0.5 ^aA^	11.5 ± 0.7 ^bcC^
2	260.7 ± 8.4 ^aC^	322.4 ± 14.8 ^bB^	445.2 ± 16.8 ^bA^	314.5 ± 10.9 ^bB^	10.6 ± 0.3 ^bD^	11.3 ± 0.5 ^bC^	13.5 ± 0.4 ^aA^	12.0 ± 0.5 ^aB^
3	262.2 ± 11.3 ^aC^	199.5 ± 14.0 ^dD^	469.4 ± 9.3 ^aA^	336.7 ± 14.9 ^aB^	10.6 ± 0.3 ^bC^	6.7 ± 0.3 ^cD^	13.1 ± 0.2 ^aA^	11.8 ± 0.3 ^abB^

^a–e^ Values in the same column with the different superscript letters denote significantly different (*p* < 0.05). ^A–D^ Values in the same row of each parameter with the different superscript letters denote significantly different (*p* < 0.05). PE, potato extract; FD-PFJ, freeze-dried potato fruit juice; PPI, potato protein isolate; EW, egg white.

**Table 3:** Breaking force and penetration distance of the Pacific whiting surimi gels with four different inhibitors (PE, FD-PFJ, PPI, and EW) at 0, 0.5, 1, 2, and 3% levels held at 60 °C for 30 min prior to heating to 90 °C.

Concentration (%)	Breaking Force (g)				Penetration Distance (mm)			
	PE	FD-PFJ	PPI	EW	PE	FD-PFJ	PPI	EW
0	43.0 ± 3.0 ^c^	43.0 ± 3.0 ^d^	43.0 ± 3.0 ^e^	43.0 ± 3.0 ^e^	1.4 ± 0.3 ^c^	1.4 ± 0.3 ^e^	1.4 ± 0.3 ^d^	1.4 ± 0.3 ^d^
0.5	43.8 ± 1.4 ^cD^	53.7 ± 4.5 ^cC^	126.7 ± 2.4 ^dA^	108.6 ± 10.7 ^dB^	1.2 ± 0.2 ^cC^	2.1 ± 0.3 ^dB^	5.9 ± 0.3 ^cA^	5.4 ± 0.6 ^cA^
1	46.0 ± 2.4 ^cD^	91.0 ± 4.9 ^bC^	211.6 ± 9.0 ^cA^	194.3 ± 11.0 ^cB^	1.5 ± 0.2 ^cC^	3.8 ± 0.3 ^cB^	8.9 ± 0.4 ^aA^	8.7 ± 0.5 ^bA^
2	62.3 ± 3.1 ^bD^	135.5 ± 15.8 ^aC^	248.2 ± 13.1 ^bB^	284.8 ± 9.9 ^bA^	2.4 ± 0.1 ^bD^	6.0 ± 0.6 ^aC^	9.8 ± 0.6 ^bB^	11.1 ± 0.5 ^aA^
3	80.8 ± 3.6 ^aD^	85.6 ± 2.5 ^aC^	266.0 ± 9.4 ^aB^	330.5 ± 8.0 ^aA^	3.3 ± 0.3 ^aC^	3.2 ± 0.2 ^bC^	9.1 ± 0.3 ^aB^	11.4 ± 0.2 ^aA^

^a–e^ Values in the same column with the different superscript letters denote significantly different (*p* < 0.05). ^A–D^ Values in the same row of each parameter with the different superscript letters denote significantly different (*p* < 0.05). PE, potato extract; FD-PFJ, freeze-dried potato fruit juice; PPI, potato protein isolate; EW, egg white.

**Table 4:** Water retention ability of the Pacific whiting surimi gels with four different inhibitors (PE, FD-PFJ, PPI, and EW) at 0, 0.5, 1, 2, and 3% levels heated to 90 °C directly (90) and held at 60 °C for 30 min prior to heating to 90 °C (60/90).

Concentration (%)	90				60/90			
	PE	FD-PFJ	PPI	EW	PE	FD-PFJ	PPI	EW
0	49.7 ± 1.5 ^b^	49.7 ± 1.5 ^a^	49.7 ± 1.5 ^b^	49.7 ± 1.5 ^bc^	26.8 ± 1.6 ^b^	26.8 ± 1.6 ^c^	26.8 ± 1.6 ^d^	26.8 ± 1.6 ^c^
0.5	66.9 ± 1.0 ^aA^	50.5 ± 2.1 ^aB^	48.3 ± 0.4 ^bB^	50.9 ± 1.3 ^bcB^	51.2 ± 2.8 ^aAB^	34.5 ± 2.0 ^bC^	46.4 ± 1.5 ^bcB^	53.4 ± 1.9 ^aA^
1	66.7 ± 2.0 ^aA^	49.5 ± 1.1 ^aB^	51.0 ± 0.4 ^bB^	53.3 ± 3.3 ^aB^	52.0 ± 4.0 ^aA^	40.9 ± 3.3 ^aB^	48.5 ± 0.6 ^bAB^	52.6 ± 2.3 ^aA^
2	65.2 ± 1.9 ^aA^	41.8 ± 1.4 ^bC^	64.4 ± 1.8 ^aA^	51.9 ± 1.0 ^abB^	54.4 ± 2.0 ^aB^	32.7 ± 1.3 ^bC^	62.3 ± 1.3 ^aA^	51.8 ± 2.4 ^aB^
3	66.2 ± 1.5 ^aA^	32.6 ± 0.5 ^cC^	47.4 ± 0.8 ^bB^	47.7 ± 2.3 ^cB^	53.0 ± 3.2 ^aA^	27.4 ± 3.1 ^cC^	42.6 ± 0.8 ^cB^	50.0 ± 1.8 ^bA^

^a–d^ Values in the same column with the different superscript letters denote significantly different (*p* < 0.05). ^A–D^ Values in the same row of each heating condition with the different superscript letters denote significantly different (*p* < 0.05). PE, potato extract; FD-PFJ, freeze-dried potato fruit juice; PPI, potato protein isolate; EW, egg white.

**Table 5:** Effect of inhibitors on the whiteness of PW surimi gels with four different inhibitors (PE, FD-PFJ, PPI, and EW) at 0, 0.5, 1, 2, and 3% levels heated to 90 °C directly (90) and held at 60 °C for 30 min prior to heating to 90 °C (60/90).

Concentration (%)	90				60/90			
	PE	FD-PFJ	PPI	EW	PE	FD-PFJ	PPI	EW
0	77.5 ± 0.6 ^a^	77.5 ± 0.6 ^d^	77.5 ± 0.6 ^b^	77.5 ± 0.6 ^c^	80.5 ± 0.3 ^a^	80.5 ± 0.3 ^bc^	80.5 ± 0.3 ^a^	80.5 ± 0.3 ^a^
0.5	76.3 ± 0.3 ^bC^	77.8 ± 0.6 ^dA^	77.3 ± 0.3 ^bB^	78.1 ± 0.6 ^bcA^	79.7 ± 0.3 ^bB^	80.2 ± 0.7 ^bcA^	79.0 ± 0.5 ^cC^	79.1 ± 0.4 ^cBC^
1	76.0 ± 0.4 ^cC^	78.5 ± 0.5 ^cA^	77.5 ± 0.2 ^bB^	77.7 ± 0.7 ^abB^	78.6 ± 0.5 ^cC^	80.1 ± 0.5 ^cA^	79.1 ± 0.3 ^cB^	78.9 ± 0.4 ^cBC^
2	74.5 ± 0.4 ^dD^	79.4 ± 0.7 ^bA^	77.2 ± 0.3 ^bC^	78.1 ± 0.4 ^abB^	77.1 ± 0.4 ^dD^	80.7 ± 0.6 ^bA^	79.3 ± 0.2 ^bcB^	79.1 ± 0.4 ^cC^
3	73.1 ± 0.3 ^eC^	80.0 ± 0.7 ^aA^	78.3 ± 0.2 ^aB^	78.3 ± 0.6 ^aB^	75.7 ± 0.4 ^eC^	81.8 ± 0.5 ^aA^	79.5 ± 0.1 ^bB^	79.7 ± 0.2 ^bB^

^a–e^ Values in the same column with the different superscript letters denote significantly different (*p* < 0.05). ^A–D^ Values in the same row of each heating condition with the different superscript letters denote significantly different (*p* < 0.05). PE, potato extract; FD-PFJ, freeze-dried potato fruit juice; PPI, potato protein isolate; EW, egg white.

## Data Availability

The data presented in this study are available on request from the corresponding author.

## Supplementary Materials

The following supporting information can be downloaded at: https://www.mdpi.com/article/10.3390/foods11193114/s1, Table S1: Effect of inhibitors on the chroma of PW surimi gels with four different inhibitors (PE, FD-PFJ, PPI, and EW) at 0, 0.5, 1, 2, and 3% levels heated to 90 °C directly (90) and held at 60 °C for 30 min prior to heating to 90 °C (60/90); Table S2: Effect of inhibitors on the hue of PW surimi gels with four different inhibitors (PE, FD-PFJ, PPI, and EW) at 0, 0.5, 1, 2, and 3% levels heated to 90 °C directly (90) and held at 60 °C for 30 min prior to heating to 90 °C (60/90).
