# Supplementary material for: Effects of Potato Protein Isolated Using Ethanol on the Gelation and Anti-Proteolytic Properties in Pacific Whiting Surimi
Source: Foods. 2022 Oct 6;11(19):3114. doi: 10.3390/foods11193114 (PMC9563566; doi:10.3390/foods11193114)
Supplement: Supplementary file 1 [file foods-11-03114-s001.zip › foods-1929873-supplementary.pdf]

Supplementary Material

The chroma and hue angle were calculated using L^*, a^*, and b^* values obtained by a colorimeter according to the following equations:

$$Chroma = (a^{*2} + b^{*2})^{1/2}$$

$$Hue\ angle = \tan^{-1}(b^*/a^*)$$

Table S1. Effect of inhibitors on the chroma of PW surimi gels with four different inhibitors (PE, FD-PFJ, PPI, and EW) at 0, 0.5, 1, 2, and 3% levels heated to 90 °C directly (90) and held at 60 °C for 30 min prior to heating to 90 °C (60/90)

Concentration (%)	90				60/90			
	PE	FD-PFJ	PPI	EW	PE	FD-PFJ	PPI	EW
0	4.2 ± 0.1^{c}	4.2 ± 0.1^{e}	4.2 ± 0.1^{e}	4.2 ± 0.1^{e}	4.4 ± 0.1^{c}	4.4 ± 0.1^{e}	4.4 ± 0.1^{e}	4.4 ± 0.1^{d}
0.5	4.1 ± 0.1^{dC}	4.6 ± 0.1^{dA}	4.6 ± 0.1d^{AB}	4.5 ± 0.2^{dB}	4.4 ± 0.1^{cB}	5.1 ± 0.2^{dA}	5.0 ± 0.1^{dA}	4.5 ± 0.1^{dB}
1	4.2 ± 0.1^{cdC}	5.2 ± 0.3^{Ca}	5.3 ± 0.1^{cA}	4.8 ± 0.2^{cB}	4.4 ± 0.1^{cD}	6.0 ± 0.4^{cA}	5.5 ± 0.2^{cB}	5.0 ± 0.1^{cC}
2	4.3 ± 0.1^{bD}	7.2 ± 0.3^{bA}	6.6 ± 0.2^{bB}	5.5 ± 0.1^{bC}	4.6 ± 0.1^{bD}	8.9 ± 0.5^{bA}	6.6 ± 0.3^{bB}	5.6 ± 0.1^{bC}
3	4.4 ± 0.1^{aD}	9.0 ± 0.6^{aA}	7.3 ± 0.1^{aB}	6.3 ± 0.1^{aC}	4.8 ± 0.1^{aD}	10.4 ± 0.4^{aA}	7.1 ± 0.2^{aB}	6.7 ± 0.1^{aC}

$^{a\text{-}e}$ Values in the same column with the different superscript letters denote significantly different ($P < 0.05$).
$^{A\text{-}D}$ Values in the same row of each heating condition with the different superscript letters denote significantly different ($P < 0.05$).
PE, potato extract; FD-PFJ, freeze-dried potato fruit juice; PPI, potato protein isolate; EW, egg white

Table S2. Effect of inhibitors on the hue of PW surimi gels with four different inhibitors (PE, FD-PFJ, PPI, and EW) at 0, 0.5, 1, 2, and 3% levels heated to 90 °C directly (90) and held at 60 °C for 30 min prior to heating to 90 °C (60/90)

**Concentration (%)**	**90**				**60/90**			
	**PE**	**FD-PFJ**	**PPI**	**EW**	**PE**	**FD-PFJ**	**PPI**	**EW**
0	158.0 ± 4.8^{a}	158.0 ± 4.8^{a}	158.0 ± 4.8^{a}	158.0 ± 4.8^{a}	147.0 ± 3.0^{a}	147.0 ± 3.0^{a}	147.0 ± 3.0^{a}	147.0 ± 3.0^{a}
0.5	159.3 ± 1.3^{aA}	143.5 ± 3.0^{bC}	143.2 ± 1.5^{bC}	150.7 ± 3.2^{bB}	145.9 ± 1.1^{aA}	135.4 ± 1.6^{bB}	132.3 ± 2.0^{bC}	144.7 ± 0.9^{aA}
1	155.1 ± 1.7^{bA}	135.2 ± 3.0^{cC}	134.4 ± 2.0^{cC}	147.2 ± 2.3^{cB}	144.1 ± 1.4^{bA}	126.4 ± 1.4^{cD}	129.2 ± 2.0^{cC}	140.9 ± 1.2^{bB}
2	149.3 ± 1.6^{cA}	$120.8 \pm 1.5d^{C}$	113.4 ± 0.6^{dD}	141.8 ± 1.0^{dB}	139.8 ± 1.7^{cA}	113.4 ± 0.8^{dC}	110.7 ± 0.7^{dD}	137.5 ± 1.7^{cB}
3	142.9 ± 1.0^{dA}	114.4 ± 1.0^{eD}	117.5 ± 0.5^{dC}	141.7 ± 0.9^{dB}	134.7 ± 0.9^{dA}	105.5 ± 0.4^{eC}	114.0 ± 0.4^{eB}	135.2 ± 0.9^{dA}

$^{a-e}$ Values in the same column with the different superscript letters denote significantly different ($P < 0.05$).
$^{A-D}$ Values in the same row of each heating condition with the different superscript letters denote significantly different ($P < 0.05$).
PE, potato extract; FD-PFJ, freeze-dried potato fruit juice; PPI, potato protein isolate; EW, egg white
